# Low peaking-phenomenon cascade high-gain observer design with LPV/LMI method

**DOI:** 10.1371/journal.pone.0307637

**Published:** 2024-09-24

**Authors:** Qi Li, Lu Duan, Guangyu Cao, Fanwei Meng

**Affiliations:** School of Control Engineering, Northeastern University at Qinhuangdao, Qinhuangdao, Hebei, China; Air University, PAKISTAN

## Abstract

To cope with the well-known peaking phenomenon and noise sensitivity in the application of the High-Gain observer, a parameter tuning method based on the LPV/LMI approach for a 2nd-order cascade observer structure is proposed in this paper. Compared to other high-gain observer methods, this method can significantly reduce the infimum of gain in the observer, thereby reducing the peak phenomenon of state estimation and the influence of measurement output noise. By transforming the observer structure into a Luenberger-like structure, the parameters of the observer can be solved by one linear matrix inequality (LMI) with a high-gain effect or a 2^n^ of LMI sets (LMIs) without a high-gain effect. Then by decomposing the nonlinear part of the system dynamics into high-dimensional and low-dimensional parts, we could solve the adjustable number $2^{j_{s}}$ of LMIs can be solved to obtain the result with limited high-gain effect. Stability analysis based on the Lyapunov method proves the convergence of this method, and the effectiveness of this method is verified through applications to one single-link mechanical arm model and a vehicle trajectory estimation application.

## 1 Introduction

Since the proposal of high-gain observers, due to the convenience of adjusting a single parameter, it has been widely used in the control of nonlinear systems [1–4]. Its convenience is reflected in its design steps. Compared to directly designing the observer parameter vector *K* for the Luenberger observer, a gain matrix *T*(*θ*) = diag(*θ*, ⋯, *θ*^*n*^) is introduced in the measurement output feedback terms in the high-gain observer dynamics, making the linear part of the error dynamics a multiple of *θ*^*n*^ to the nonlinear part. Therefore, after the stability of the linear part of the observer, the stability of its nonlinear part can be achieved by adjusting the gain coefficient *θ* alone, and increasing this parameter can improve the convergence rate of the error [5]. However, to ensure the robustness of the error steady state, the gain coefficient *θ* and its related feedback gain are often too large, leading to the peaking phenomenon of measurement and high sensitivity to measurement noise, which would reduce the robustness of the observer [6].

In the past few decades, improvements for high-gain observers can be mainly classified into three categories:

1) Feedback term processing

Most improvement methods usually directly handle the excessive feedback terms caused by the gain matrix *T*(*θ*). In the feedback term processing of the observer dynamics, existing methods often improve the performance of the observer by introducing filtering, dead zone, saturation, and other processes. Treangle et al. [7] filtered the measurement output to reduce the impact of high-frequency noise. Cocetti et al. [8] achieved the same effect by introducing a dead zone into the feedback terms. Astolfi et al. [9] introduced a saturation element into the feedback terms to suppress the peak phenomenon. A similar improvement of the standard high-gain observer was proposed by Farza et al. [6]. Although adjusting the feedback items has become a convention, the information loss caused by dead zones is unacceptable in some cases [8]. While the saturated application can eliminate peak phenomena, it cannot handle the measurement sensitivity issues brought by high gain. The grid filtering technique is quite an efficient way to overcome the measurement noise, but it would cause high-frequency phase-shift that causes distortions of the estimate [10].

2) Cascade observer structure

Astolfi et al. [5] proposed a Marconi/Astolfi high-gain observer structure, observing an n-dimensional system through the cascade of n-1 second-order high-gain observers, limiting the order of gain to 2 while enhancing the ability to suppress high-frequency noise [11]. Meanwhile, based on this method, Khalil [12] proposed a cascade method for (n-1)th 1-dimensional sub-observers and introduced saturation in the sub-observers to reduce the peak phenomenon. Boizot et al. [13] introduced the high-gain method into the Kalman filter, under the smallest high-gain action possible to ensure the robustness of the state estimation results and the convergence of the high-gain observer. The cascade structures proposed above do have the ability to cope with the measurement noise, but the actual gain to measurement noise in the higher dimensional is the same as the standard high-gain observer.

3) Optimization of the gain coefficient

Alessandri et al. [14] proposed a standard high-gain observer structure with increasing gain *θ* over time to deal with the peaking phenomenon at the start of the convergence of state estimation. Zemouche et al. [15] combined a method called LPV/LMI [16] with the design of high-gain observers to propose a new low-gain observer design method, which can reduce the dependence of the stability of the nonlinear part on the gain coefficient by optimizing the observer’s linear part, i.e., the value of the observer coefficient *K*, thereby reducing the overall gain of the observer while stabilizing the system. The utilization of the gain optimization technique is a convenient way to balance the performance of the high-gain effect and the robustness of the observer. Thus, the time-varying gain or gain with a lower limit bound could also be utilized on the other observer structures, which is mentioned in [15].

Based on the above research, this paper proposes a parameter design method based on LPV/LMI technology for Marconi/Astolfi type observer structures. Compared to directly designing parameters for the standard high-gain observer in [15], this method can reduce sensitivity to measurement noise and, compared to [5], maintains low gain characteristics. First, the observer structure needs to be transformed into a canonical Luenberger form, and then the single LMI solution method corresponding to the pole configuration is obtained. Then, the LPV/LMI form of the multiple LMIs solution method is obtained through the gradient decomposition of the nonlinear characteristics. Finally, the parameter solution algorithm is given, and the simulation effect is compared through two examples of a single-link mechanical arm and a vehicle trajectory estimation.

The innovations of this work are as follows:

- By converting the error dynamic into the Luenberger-like form, we develop a parameter design method for the 2nd-order cascade observer structure [5] based on the LMI and LPV/LMI method and prove the stability (Section 3.1 and Section 3.2).
- Based on the LPV/LMI methods, the nonlinear part could be decomposed in the low dimensional and high dimensional parts by grid decomposition and processed separately with high gain effect and LPV/LMI methods, and finally obtain the main theorem. Then we propose an algorithm to calculate the parameter(Section 3.3).
- Two examples are used to verify the practicality of the proposed method in this paper. By comparing our method with the original pole assign method [5], the standard High-gain observer method [1], and a new filtered High-gain observer [7], the effectiveness of our method has been proved.

Notations:

- (*A*_*i*_, *B*_*i*_, *C*_*i*_) present the observable canonical triplets of dimension *i*,
  $$\begin{array}{c} \begin{array}{c} A_{i}={\left[\begin{array}{ccccc} 0 & 1 & 0 & \cdots & 0\\ 0 & 0 & 1 & \cdots & 0\\ \vdots & \vdots & \vdots & \ddots & \vdots \\ 0 & 0 & 0 & \cdots & 1\\ 0 & 0 & 0 & \cdots & 0 \end{array}\right]}_{i\times i},\;B_{i}={\left[\begin{array}{c} 0\\ \vdots \\ 0\\ 1 \end{array}\right]}_{i\times 1},\\ C_{i}={\left[\begin{array}{cccc} 1 & 0 & \cdots & 0 \end{array}\right]}_{1\times i}. \end{array} \end{array}$$
- *I*_*i*_ denotes the identity
-
$L_{f}h\left(x\right)=\frac{\partial h}{\partial x}\cdot f$ is the Lie derivative of *h*(*x*) under vector field *f*.
- *T*_*i*_(*θ*) = *diag*(*θ*, *θ*^2^, ⋯, *θ*^*i*^).
-
$D_{1}=\left[\begin{array}{cc} -1 & 0\\ -1 & 0 \end{array}\right]$ , $D_{2}=\left[\begin{array}{cc} 0 & 1\\ 0 & 1 \end{array}\right]$.
-
$He\left(Q\right)=Q+Q^{⊤}$ .

## 2 Problem description

### 2.1 System description

We consider a class of nonlinear single input single output (SISO) systems of the form

$$\begin{array}{c} \left\{ \begin{array}{c} \dot{z}=f\left(z\right)+\bar{g}\left(z\right)u+\bar{d}\\ y=h(z)+v \end{array}\right. \end{array}$$ (1)

where $z\in R^{m}$ is the state variable, y∈R is the measured output, u∈R is the input, *f*(⋅), *g*(⋅) and *h*(⋅) are *C*^∞^ functions. $\bar{d}\in R^{m}$ and v∈R represent the system disturbance and measurement disturbance, respectively. Both disturbances are bounded.

Let *Z*_*u*_(*z*_0_, *t*) be the solution of (1) going through *z*_0_ at time 0 with input *u*. The definition of observability of nonlinear system (1) that will be crucial for the following analysis is reviewed.

**Definition 1** (Differential Observability). [17] *System* (1) *is differentially observable of order N on an open subset* $S\in R^{m}$, *if mapping* $H_{N}\left(z\right)={\left[\begin{array}{cccc} h(z) & L_{f}h\left(z\right) & \cdots & L_{f}^{N-1}h\left(z\right) \end{array}\right]}^{⊤}$ *is injective on* S. *Furthermore, it is regarded as strongly differentially observable on* S *if the mapping is also an immersion*.

**Definition 2** (Uniform Observability). [17] *System* (1) *is uniformly observable on an open subset* $S\in R^{m}$, *if for any pairs* $\left(x_{a},x_{b}\right)\in S^{2}$ *with x*_*a*_ ≠ *x*_*b*_, *any T* > 0, *and any* *C*^1^ *input u defined on* [0, *T*), *there exists a time t* < *T* *such that h*(*X*_*u*_(*x*_*a*_, *t*)) ≠ *h*(*X*_*u*_(*x*_*b*_, *t*)) *and* $\left(X_{u}\left(x_{a},s\right),X_{u}\left(x_{b},s\right)\right)\in S^{2}$ *for all* *s* ≤ *t*.

**Lemma 1**. [18] *If system* (1) *is uniformly observable and strongly differentially observable of order N* = *m on an open set* S *containing the compact set* Z, *it can be transformed on* Z *into a full Lipschitz triangular canonical form of dimension n* = *m*.

In general, if system (1) is uniformly observable and 2-order strongly differentially observable, as stated in Lemma 1, by selecting *x* = *H*_*n*_(*z*), we can obtain

$$\begin{array}{c} \left\{ \begin{array}{c} \dot{x}=A_{n}x+B_{n}\varphi \left(x\right)+g\left(x\right)u+d\\ y=C_{n}x \end{array}\right. \end{array}$$ (2)

which is the actual observed system, where $x\in X\in R^{n}$, *φ*(*x*) can be chosen satisfying $\varphi \left(x\right)=L_{f}^{n}h\left(z\right)$, and $g\left(x\right)=L_{\bar{g}}H_{n}\left(x\right)={\left[g_{1}\left(h\left(z\right)\right)\cdots g_{n}(h\left(z\right),\cdots ,L_{f}^{n-1}h\left(z\right))\right]}^{⊤}$, both *φ*(⋅) and *g*(⋅) can be locally lipschitz on X as a result of Lemma 1.

**Remark 1**. *Lemma 1 ensure the existence of the canonical observability form and the Lipschitz property of nonlinear dynamics, which is a necessary condition in the designing of high-gain observers* [19].

### 2.2 Observer form

To estimate the states of (2), we use a kind of 2-order cascade high-gain observer form proposed in [5] as below

$$\begin{array}{cc} {\dot{\zeta }}_{1}= & A_{2}\zeta_{1}+B_{2}B_{2}^{⊤}\zeta_{2}+K_{1}T_{2}\left(\theta \right)(y-C_{2}\zeta_{1})\\ {\dot{\zeta }}_{i}= & A_{2}\zeta_{i}+B_{2}B_{2}^{⊤}\zeta_{i+1}+K_{i}T_{2}\left(\theta \right)(B_{2}^{⊤}\zeta_{i-1}-C_{2}\zeta_{i})\;i=2,\cdots ,n-2\\ {\dot{\zeta }}_{n-1}= & A_{2}\zeta_{n-1}+B_{2}\varphi_{s}\left(\hat{x}\right)+K_{n-1}T_{2}\left(\theta \right)(B_{2}^{⊤}\zeta_{n-2}-C_{2}\zeta_{n-1}) \end{array}$$ (3)

with

$$\begin{array}{cc} \hat{x} & =\bar{C}\zeta \\ \bar{C} & =\text{diag}\left(C_{2},\cdots ,C_{2},I_{2}\right)\in R^{(2n-2)\times n} \end{array}$$

**Fig 1:**
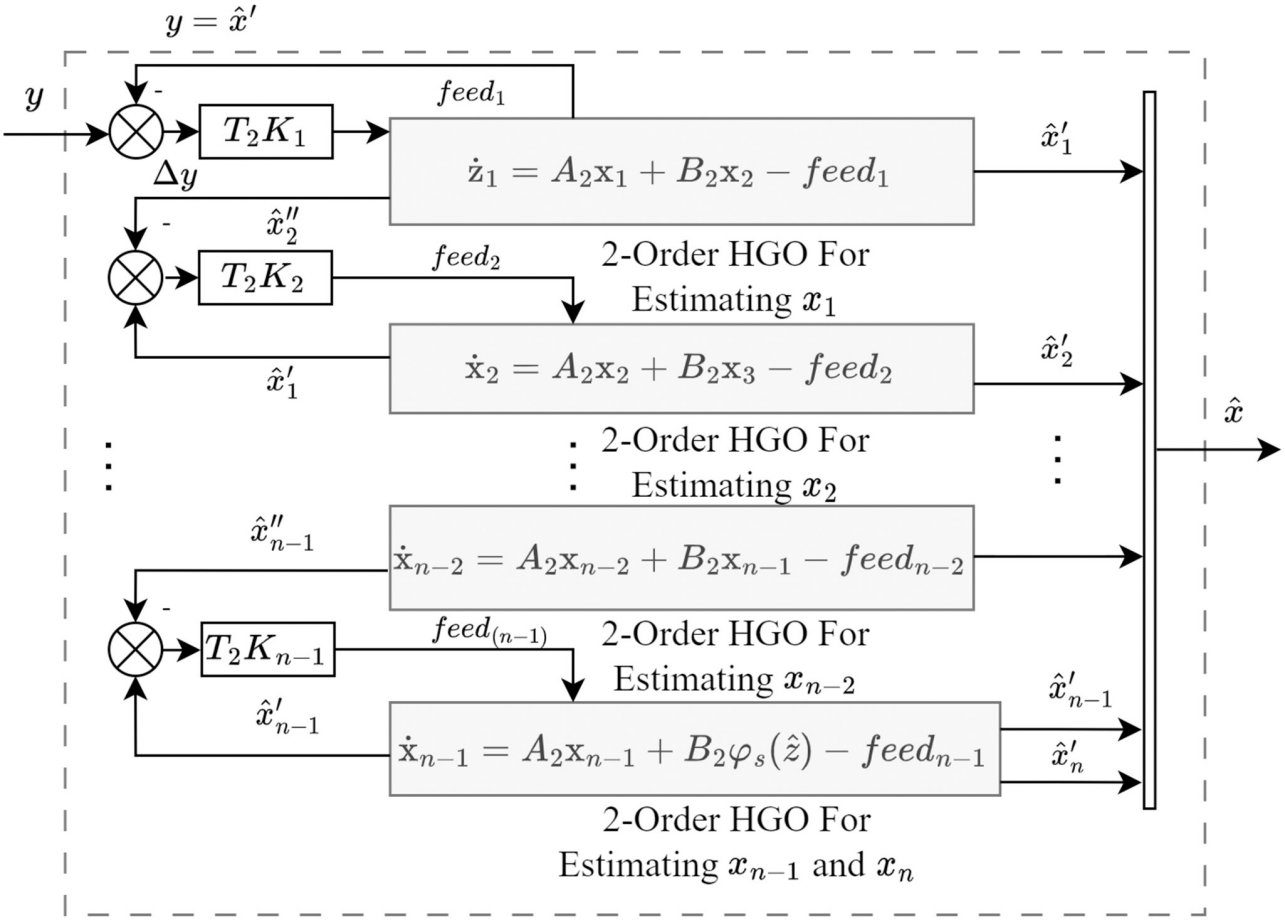
Low peaking cascade high-gain observer strucutre.

In (3), $\zeta_{i}={\left[{\hat{x}}_{i},{\hat{x}}_{i+1}^{\prime }\right]}^{⊤}$ is the state of *i*th sub-observer for *i* = 1, ⋯, *n* − 2, $\zeta_{n-1}=\left[{\hat{x}}_{n-1},{\hat{x}}_{n}\right]$ is the state of (*n* − 1)th sub-observer, ${\hat{x}}_{i}$ is the *i*th state estimation of system (2) and ${\hat{x}}_{i+1}^{\prime }$ presents the auxiliary estimation of (*i* + 1)th state, *K*_*i*_ = [*k*_*i*1_, *k*_*i*2_]^⊤^ is denoted as the design parameter of *i*th sub-observer. *φ*_*s*_(⋅) is equivalent to *φ*(⋅) in X. In this context, we assume that *φ*_*s*_(⋅) agrees with the global Lipschitz condition as below. This structure can be conceptualized as being composed of (*n* − 1) second-order high-gain sub-observers shown in Fig 1.

**Assumption 1** (Globally Lipschitz condition)

$$\begin{array}{c} |\varphi_{s}\left(x^{1}\right)-\varphi_{s}\left(x^{2}\right)|\leq L_{\varphi_{s}}\left\|x^{1}-x^{2}\right\|,\;\forall x^{1},x^{2}\in R^{n} \end{array}$$ (4)

*where* $L_{\varphi_{s}}$ *is the Lipschitz constant*.

Define **x**_*i*_ = [*x*_*i*_, *x*_*i*+1_]^⊤^ (where *x*_*i*_ is the i-th state of the system (2), then the object system (2) can be extended into following form

$$\begin{array}{c} \left\{ \begin{array}{c} {\dot{x}}_{1}=A_{2}x_{1}+B_{2}B_{2}^{⊤}x_{2}\\ {\dot{x}}_{i}=A_{2}x_{i}+B_{2}B_{2}^{⊤}x_{i+1},\;i=2,\cdots ,n-2\\ {\dot{x}}_{n-1}=A_{2}x_{n-1}+B_{2}\varphi \left(x\right) \end{array}\right. \end{array}$$

Thus we can obtain error dynamic by defining estimation error $e_{i}={\hat{x}}_{i}-x_{i}$ and auxiliary error $\epsilon_{i}={\hat{x}}_{i}^{\prime }-x_{i}$. For convenience, define state error **e** = (**e**_1_, ⋯, **e**_*n*−1_)^⊤^, where **e**_*i*_ = (*e*_*i*_, *ϵ*_*i*+1_)^⊤^. Then the error dynamic is also equivalent to

$$\begin{array}{c} \dot{e}=Me+B_{2n-2}\Delta_{\varphi }\left(\hat{x},x\right) \end{array}$$ (5)

where $\Delta_{\varphi }\left(\hat{x},x\right)=\varphi_{s}\left(\hat{x}\right)-\varphi \left(x\right)$, and system matrix is in form of

$$\begin{array}{c} M=\left[\begin{array}{cccccc} E_{1} & N & 0 & 0 & \cdots & 0\\ Q_{2} & E_{2} & N & 0 & \cdots & 0\\ 0 & Q_{3} & E_{3} & N & & 0\\ \vdots & & \ddots & \ddots & \ddots & \vdots \\ 0 & \cdots & \cdots & Q_{n-2} & E_{n-2} & N\\ 0 & \cdots & \cdots & 0 & Q_{n-1} & E_{n-1} \end{array}\right] \end{array}$$

with *E*_*i*_ = *A*_*i*_ − *T*_2_(*θ*)*K*_*i*_*C*_2_, $Q_{i}=T_{2}\left(\theta \right)K_{i}B_{2}^{⊤}$ and $N=B_{2}B_{2}^{⊤}$. Astolfi et.al. developed a pole assign method to make *M* Hurwitz in [5], as the following Lemma.

**Lemma 2**. [5] *Let* $P\left(\lambda \right)=\lambda^{2n-2}+m_{1}\lambda^{2n-3}+\cdots +m_{2n-2}$ *be an arbitrary Hurwitz polynomial. There exists a choice of* (*k*_*i*1_, *k*_*i*2_), *i* = 1, ⋯, *n* − 1 *such that the characteristic polynomial of* *M* *coincides with* P(λ).

Although estimation error can asymptotically converge by setting the gain parameter *θ* to a sufficiently large value in [5], the peaking phenomena are still present in the observation results due to the large observer gain.

In this paper, we adopt the observer form proposed in [5]. However, unlike the pole design method described in Lemma 2, observer parameter *K*_*i*_ can be optimized via multiple linear matrix inequations (LMIs) after transformation to the error dynamics, resulting in a significant reduction on the gain parameter *θ*.

## 3 Main result

First, rewrite the error dynamics (6) as below

$$\begin{array}{c} \dot{e}=\left(H+\bar{T}\left(\theta \right)LS\right)e+B_{2n-2}\Delta_{\varphi }\left(\hat{x},x\right) \end{array}$$ (6)

where

$$H=\left[\begin{array}{ccccc} A_{2} & B_{2}B_{2}^{⊤} & O & \cdots & O\\ O & A_{2} & B_{2}B_{2}^{⊤} & \cdots & O\\ \vdots & \vdots & \ddots & \ddots & \vdots \\ O & O & \cdots & A_{2} & B_{2}B_{2}^{⊤}\\ O & O & \cdots & O & A_{2} \end{array}\right]$$

$$S=\left[\begin{array}{ccccc} D_{1} & O & \cdots & \cdots & O\\ D_{2} & D_{1} & \cdots & \cdots & O\\ \vdots & \ddots & \ddots & & \vdots \\ \vdots & & D_{2} & D_{1} & O\\ O & \cdots & O & D_{2} & D_{1} \end{array}\right]$$

$$\bar{T}\left(\theta \right)=\text{diag}(T_{2}\left(\theta \right),\cdots ,T_{2}\left(\theta \right))$$

$$L=\text{diag}(L_{1},L_{2},\cdots ,L_{n-1})$$

with $L_{i}=\left[\begin{array}{cc} l_{11} & l_{12}\\ l_{21} & l_{22} \end{array}\right]$ being the parameter matrix that needs to be solved. The observer parameter matrix can be calculated as *K*_*i*_ = *L*_*i*_**1**_2×1_.

Then, transform the error variable in (6) with $e=\theta^{-1}\left(T_{n-1}\left(\theta \right)⊗T_{2}\left(\theta \right)\right)\tilde{e}$ (which is equivelent to ${\tilde{e}}_{i}={\left[\begin{array}{cc} \theta^{-i}e_{i} & \theta^{-(i+1)}\epsilon_{i+1} \end{array}\right]}^{⊤}$). And we have the following transformed error dynamic.

$$\begin{array}{c} \dot{\tilde{e}}=\theta \left(H+LS\right)\tilde{e}+\theta^{-n}B_{2n-2}\Delta_{\varphi }\left(\hat{x},x\right) \end{array}$$ (7)

**Remark 2**. *Since we are using the transformed error dynamics* $\tilde{e}$, *the nonlinear part of the error dynamics here is* $\theta^{-n}B_{2n-2}\Delta_{\varphi }\left(T_{n}\tilde{e}\right)$. *By the mean value theorem, its linearized result is* $\theta^{-n}B_{2n-2}T_{n}\nabla \tilde{e}$, *where* ∇ *is the gradiant of* *φ*_*s*_. *From the definition of the high-gain matrix* *T*_*n*_, *it can be seen that higher-dimensional nonlinear gradients are less affected by high gains, which ultimately leads to our main result*.

*Under the new form of error dynamics in* (7), *the value of K*_*i*_ *can be determined using an LMI-based method rather than relying solely on pole assignment, which corresponds to Theorem 1. The high-gain matrix T*_*n*_ *is used to handle the nonlinear components. Then by decomposing the nonlinear error* $\Delta_{\varphi }\left(\hat{x},x\right)$ *into gradients, the nonlinear constraints are transformed into a set of* 2^*n*^ *linear constraints. This allows us to use multiple LMIs to address both the nonlinear components and the pole assignment for the observer, which is proved in Theorem 2*.

*Finally, we decompose the nonlinear constraint into two linear constraint sets based on dimensionality. The low-dimensional component is handled using the approach from Theorem 1 with high gains, while the high-dimensional component is tackled using the LPV/LMI method outlined in Theorem 2. Ultimately, this process leads to the formulation of the theorem and algorithm presented in Theorem 2*.

Under the new form of error dynamics in (7), the value of *K*_*i*_ can be determined using an LMI-based solver method rather than relying on pole assignment.

### 3.1 1-LMI optimization with the largest infimum of gain

This approach is presented and supported by the following theorem.

**Theorem 1**. *Consider the error dynamics* (6). *The state estimation error e will be asymptotically stable if there exist scalar* *μ* ∈ (0, 1), *and matrices* $R_{i}\in R^{2\times 2}$ *for* *i* = 1, ⋯, *n* − 1, *and symmetric positive definite matrices* $P_{i}\in R^{2\times 2}$ *for* *i* = 1, ⋯, *n* − 1, *such that the following LMI is feasible*:

$$\begin{array}{c} H^{⊤}P+PH+S^{⊤}R^{⊤}+RS+\mu I_{2n-2}<0 \end{array}$$ (8)

*where*

$$P=\text{diag}(P_{1},\cdots ,P_{n-1})$$

$$R=\text{diag}(R_{1},\cdots ,R_{n-1})$$

*Once the LMIs are solved*, *L* = *P*^−1^*R*, *K* = *L* **1**_(2*n*−2)×1_, *and* $\theta \in \{\theta |\theta \geq \theta_{0},\theta_{0}=\text{min}\;(3L_{\varphi_{s}}\left\|P\right\|/\mu ,2)\}$.

*Proof*. Choose Lyapunov function as $V\left(\tilde{e}\right)={\tilde{e}}^{⊤}P\tilde{e}$, then

$$\begin{array}{cc} \dot{V} & ={\dot{\tilde{e}}}^{⊤}P\tilde{e}+{\tilde{e}}^{⊤}P\dot{\tilde{e}}\\ & =He({\left(\theta \left(H+RS\right)e+\theta^{-n}B_{2n-2}\Delta_{\varphi }\left(\hat{x},x\right)\right)}^{⊤}P\tilde{e}) \end{array}$$ (9)

Notice that nonlinear error $\Delta_{\varphi }\left(\hat{x},x\right)$ can be transformed into time-variable gradient form

$$\begin{array}{cc} \Delta_{\varphi_{s}}\left(\hat{x},x\right) & =\varphi_{s}\left(\hat{x}\right)-\varphi_{s}\left(x\right)\\ & =\nabla_{\varsigma }\left(\hat{x}-x\right)\\ & =\nabla_{\varsigma }T_{n}\left(\theta \right)Ce \end{array}$$ (10)

where $\nabla_{\varsigma }=\left[\begin{array}{ccc} \frac{\partial \varphi_{s}}{\partial x_{1}}{|}_{\varsigma } & \cdots & \frac{\partial \varphi_{s}}{\partial x_{n}}{|}_{\varsigma } \end{array}\right]$ denotes the gradient of *φ*_*s*_(*x*) at *x* = *ς*. *ς* refers to a point between *x* and $\hat{x}$, and $\bar{C}=\text{diag}\left(C_{2},\cdots ,C_{2},I_{2}\right)$.

For simplicity, we will replace the expression $\frac{\partial \varphi_{s}}{\partial x_{i}}{|}_{\varsigma }$ with $\frac{\partial \varphi_{s}}{\partial x_{i}}$, and utilize ∇ instead of ∇_*ς*_.

By substituting (8) and (10) in (9), following inequality is obtained

$$\begin{array}{cc} \dot{V} & =He({\left(\theta \left(H+RS\right)e+\theta^{-n}B_{2n-2}\nabla T_{n}\left(\theta \right)Ce\right)}^{⊤}P\tilde{e})\\ & \leq \theta \tilde{e}\{He(-\frac{1}{2}\mu I_{2n-2}+\theta^{-1-n}PB_{2n-2}\nabla T_{n}\left(\theta \right)\bar{C})\}\tilde{e} \end{array}$$ (11)

Let *ϑ* = ‖*P*‖^−1^*θ*, then if ‖*P*‖ ≥ 1, there is

$$\begin{array}{cc} \dot{V} & \leq \theta \tilde{e}He\{-\frac{\mu }{2}I_{2n-2}+\theta^{-1-n}PB_{2n-2}\nabla T_{n}\left(\theta \right)\bar{C}\}{\tilde{e}}^{⊤}\\ & \leq \tilde{e}He\{-\frac{\mu }{2}I_{2n-2}+\vartheta^{-1-n}{\left\|P\right\|}^{-1}PB_{2n-2}\nabla T_{n}\left(\vartheta \right)\bar{C}\}{\tilde{e}}^{⊤}\\ & \leq \tilde{e}He\{-\frac{\mu }{2}I_{2n-2}+\vartheta^{-1-n}B_{2n-2}\nabla T_{n}\left(\vartheta \right)\bar{C}\}{\tilde{e}}^{⊤} \end{array}$$ (12)

According to Assumption 1, the gradient of *φ*_*s*_(*x*) is bounded, specifically satisfying $\|\nabla_{x}\varphi_{s}{\left(x\right)\|}_{\infty }\leq L_{\varphi_{s}}$. This implies that the absolute value of each partial derivative $\frac{\partial \varphi_{s}\left(x\right)}{\partial x_{i}}$ is also bounded, ensuring $|\frac{\partial \varphi_{s}\left(x\right)}{\partial x_{i}}|\leq L_{\varphi }$ for all *i* = 1, ⋯, *n*. Thus we can obtain the following inequality:

$$\begin{array}{cc} & He\{-\frac{\mu }{2}I_{2n-2}+\vartheta^{-1-n}\nabla T_{n}\left(\vartheta \right)\bar{C}\}\\ \leq & \left[\begin{array}{ccccc} -\mu & 0 & \cdots & 0 & \vartheta^{-n}L_{\varphi }\\ 0 & -\mu & \cdots & 0 & 0\\ \vdots & \vdots & \ddots & \vdots & \vdots \\ 0 & 0 & \cdots & -\mu & \vartheta^{-2}L_{\varphi }\\ \vartheta^{-n}L_{\varphi } & 0 & \cdots & \vartheta^{-2}L_{\varphi } & -\mu +2\vartheta^{-1}L_{\varphi } \end{array}\right] \end{array}$$ (13)

If $\vartheta >\frac{3L_{\varphi }}{\mu }$, then

$$-\mu +2\vartheta^{-1}L_{\varphi }\leq -\vartheta^{-1}L_{\varphi }$$

Meanwhile, since *θ* > 2, it follows that

$$\left|-\mu +2\vartheta^{-1}L_{\varphi }|>|\vartheta^{-1}L_{\varphi }|>\sum_{i=2}^{n}|\vartheta^{-i}L_{\varphi }\right|$$

and

$$\mu >\vartheta^{-i}L_{\varphi }$$

which means that $He\{-\frac{\mu }{2}I_{2n-2}+\vartheta^{-1-n}\nabla T_{n}\left(\vartheta \right)\bar{C}\}$ exhibits diagonal dominance, which, as per Gershgorin’s circle Theorem [20], implies it is negative definite. Thus we have

$$\dot{V}<0$$

Consequently, $V(\tilde{e})$ asymptotically decreases towards zero, indicating the asymptotic stability of the transformed estimation error $\tilde{e}$.

Furthermore, since the actual estimation error *e* is linearly related to $\tilde{e}$, the asymptotic stability of estimation error *e* is obtained.

In other cases where ‖*P*‖ ≤ 1, the same result can be obtained by selecting *ϑ* = *θ* directly. Thus the proof is finished.

Theorem 1 presents a method for optimizing the observer parameter matrix *K*. However, similar to the pole assign method, the gain of the observer can become significantly large, leading to a pronounced peaking phenomenon in the estimation results. In contrast, by employing the LMI technique, we can tune the observer parameter by solving multiple LMIs in the following context. This allows us to mitigate or even eliminate the high-gain effect, resulting in improved estimation performance.

### 3.2 2^*n*^-LMIs optimization with minimum infimum of gain

Assumption 1 implies that the gradient ∇ is bounded within a compact set Φ, given by:

$$\Phi =\{\left[\phi_{1},\cdots ,\phi_{n}\right]|\phi_{i}\in \left[-L_{\varphi_{s}},L_{\varphi_{s}}\right]\}$$

where *ϕ*_*i*_ represents the partial derivative ∂*φ*_*s*_/∂*x*_*i*_. It’s a compact set with its vertices contained in the set:

$$V_{\Phi }=\{{\left[\phi_{1},\cdots ,\phi_{n}\right]}^{⊤}|\phi_{i}\in \left\{-L_{\varphi_{s}},L_{\varphi_{s}}\right\}\;\forall i=1,\cdots ,n\}$$

Consequently, the nonlinear error $\Delta_{\varphi_{s}}\left(\hat{x},x\right)$ belongs to the set $\left(\Phi T_{n}\left(\theta \right)\bar{C}e\right)$, and the Lyapunov derivative (9) can also be contained within a set related to Φ as below:

$$\begin{array}{cc} \dot{V} & \in \theta {\tilde{e}}^{⊤}\{He(PH+RS+\theta^{-1-n}PB_{2n-2}\Phi T_{n}\left(\theta \right)\bar{C})\}\tilde{e}\\ & =\theta {\tilde{e}}^{⊤}\{He(P(H+\theta^{-1-n}B_{2n-2}\Phi T_{n}\left(\theta \right)\bar{C})+RS)\}\tilde{e}\\ & =\theta {\tilde{e}}^{⊤}\{He(PH(\Phi )+RS)\}\tilde{e} \end{array}$$ (14)

where $H\left(\Phi \right)=H+\theta^{-1-n}B_{2n-2}\Phi T_{n}\left(\theta \right)\bar{C}$.

By treating the Lipschitz nonlinear dynamic as a Linear Parameter-Variable (LPV) part, it is possible to utilize multiple Linear Matrix Inequalities (LMIs) techniques to solve the observer parameter matrix *K* without resorting to the high-gain method (And the gain parameter *θ* = 1 in this situation). This approach allows for achieving stability in the presence of the dominant linear component while considering the Lipschitz nonlinearity separately. Thus we have the following Theorem:

**Theorem 2**. *Consider the error dynamic* (6). *Let θ*_0_ *represent the infimum of the gain. The state estimation error e will be asymptotically stable if there exists scalar μ* ∈ (0, 1) *and, matrices* $R_{i}\in R^{2\times 2}$ *for* *i* = 1, ⋯, *n* − 1, *and symmetric positive definite matrices* $P_{i}\in R^{2\times 2}$ *for i* = 1, ⋯, *n* − 1, *such that the following* 2^*n*^ *Linear Matrix Inequalities (LMIs) are feasible*:

$$\begin{array}{c} H{\left(\nu_{i}\right)}^{⊤}P+PH\left(\nu_{i}\right)+S^{⊤}R^{⊤}+RS<-\mu I_{2n-2},\;\forall \nu_{i}\in V_{\Phi } \end{array}$$ (15)

*where*

$$H\left(\nu_{i}\right)=H+\theta^{-1-n}B_{2n-2}\nu_{i}T_{n}\left(\theta \right)\bar{C}$$

$$P=\text{diag}(P_{1},\cdots ,P_{n-1})$$

$$R=\text{diag}(R_{1},\cdots ,R_{n-1})$$

*Once the LMIs are solved*, *L* = *P*^−1^*R*, *K* = *L* **1**_2*n*−2×1_, *and* *θ* ∈ {*θ*|*θ* ≥ *θ*_0_, *θ*_0_ = 1}.

*Proof*. Given that Φ is a compact set with vertices $\nu_{i}\in V_{\Phi }$, it follows that for every ∇ ∈ Φ, the following feature is satisfies:

$$\nabla \leq \sum_{i=1}^{2^{n}}p_{i}\nu_{i},\;\sum_{i=1}^{2^{n}}p_{i}=1,\;p_{i}\geq 0$$

thus

$$\begin{array}{cc} H(\nabla ) & =H+\theta^{-1-n}B_{2n-2}\nabla T_{n}\left(\theta \right)\bar{C}\\ & \leq \sum_{i=1}^{2^{n}}p_{i}(H+\theta^{-1-n}B_{2n-2}\nu_{i}T_{n}\left(\theta \right)\bar{C})\\ & =\sum_{i=1}^{2^{n}}p_{i}H\left(\nu_{i}\right) \end{array}$$

Let *θ* = 1, then the following inequality is satisfied for (9)

$$\begin{array}{cc} \dot{V}\left(\nabla \right) & \leq {\tilde{e}}^{⊤}\{He(\sum_{i=1}^{2^{n}}p_{i}PH\left(\nu_{i}\right)+RS)\}\tilde{e}\\ & =\sum_{i=1}^{2^{n}}p_{i}{\tilde{e}}^{⊤}\{He(PH(\nu_{i})+RS)\}\tilde{e} \end{array}$$

substituting (15) into it, then we have

$$\dot{V}\left(\nabla \right)<0$$

Finally, following the proof outlined in Theorem 1, we could establish the asymptotic stability of the state estimation error *e*.

Theorem 2 presents an alternative approach to determining the observer parameter, *K*, that eliminates the need for considering the gain effect. However, it may encounter challenges when applying this method, particularly in cases where the Lipschitz constant is large. The optimization process requires solving a significant number of LMIs, resulting in constraints that vary significantly. This burdens the LMI solver considerably and may even render the task infeasible due to computational limitations.

Thus in the next section, by combining Theorem 1 and Theorem 2, the main result is achieved through the solution of a variable number of multiple LMIs, enabling the determination of the observer parameter *K*. Moreover, this approach ensures that the infimum of gain required is sufficiently modest.

### 3.3 $2^{j_{s}}$-LMIs optimization with limited infimum of gain

According to (13), it can be observed that the impact of the gain *θ* on the nonlinear component diminishes as the state order increases. This implies that the high-gain effect is more pronounced in lower-order states compared to higher-order ones.

It is natural to decompose the nonlinear error $\Delta_{\varphi_{s}}\left(\hat{x},x\right)$ into lower-order and higher-order components:

$$\begin{array}{cc} \Delta_{\varphi }\left(x,\hat{x}\right) & =\left[\begin{array}{ccc} \frac{\partial \varphi_{s}}{\partial x_{1}} & \cdots & \frac{\partial \varphi_{s}}{\partial x_{n}} \end{array}\right]e\\ & =(\nabla_{HG}+\nabla_{LMI})e\\ & =\nabla_{HG}e_{\left[1,n-j_{s}\right]}+\nabla_{LMI}e_{\left[n-j_{s}+1,n\right]} \end{array}$$ (16)

where *e*_[*a*,*b*]_ = [0, ⋯, 0, *e*_*a*_, ⋯, *e*_*b*_, 0, ⋯, 0]^⊤^ for *b* > *a*, $\nabla_{HG}=\left[\begin{array}{cccccc} \frac{\partial \varphi_{s}}{\partial x_{1}} & \cdots & \frac{\partial \varphi_{s}}{\partial x_{n-j_{s}}} & 0 & \cdots & 0 \end{array}\right]$ represents the lower-order components for high-gain effect, and $\nabla_{LMI}=\left[\begin{array}{cccccc} 0 & \cdots & 0 & \frac{\partial \varphi_{s}}{\partial x_{n-j_{s}+1}} & \cdots & \frac{\partial \varphi_{s}}{\partial x_{n}} \end{array}\right]$ denotes the higher-order components associated with the LMIs effect, thus ∇ = ∇_*HG*_ + ∇_*LMI*_.

This allows us to independently apply the high-gain effect and the LMIs effect to each component. By doing so, we can reduce both the number of required LMIs and the infimum of gain *θ*.

Take $I_{\left[j_{s}-1,n\right]}=\text{diag}(I_{n-j_{s}},O_{j_{s}})$. Additionally, we define a set $\Phi^{j_{s}}=\{\left[0,\cdots ,0,\phi_{n-j_{s}+1},\cdots ,\phi_{n}\right]|\phi_{i}\in \left[-L_{\varphi },L_{\varphi }\right]\}$, and consider the vertices of this set denoted by

$$V_{\Phi }^{j_{s}}=\{\left[0,\cdots ,0,\phi_{n-j_{s}+1},\cdots ,\phi_{n}\right]|\phi_{i}\in \{-L_{\varphi },L_{\varphi }\}\}$$

then we have the following main Theorem.

**Theorem 3**. *Consider the error dynamic* (6). *Let θ*_0_ *represent the infimum of the gain. The state estimation error e will be asymptotically stable if there exists scalar* *μ* ∈ (0, 1), *integer* *j*_*s*_ ∈ {1, 2, ⋯, *n*}, *and matrices* $R_{i}\in R^{2\times 2}$ *for* *i* = 1, ⋯, *n* − 1, *and symmetric positive definite matrices* $P_{i}\in R^{2\times 2}$ *for* *i* = 1, ⋯, *n* − 1, *such that the following* $2^{j_{s}}$ *Linear Matrix Inequalities (LMIs) are feasible*:

$$\begin{array}{c} H{\left(\nu_{i}\right)}^{⊤}P+PH\left(\nu_{i}\right)+S^{⊤}R^{⊤}+RS<-\mu I_{2n-2},\;\forall \nu_{i}\in V_{\Phi }^{j_{s}} \end{array}$$ (17)

*where*

$$H\left(\nu_{i}\right)=H+\theta^{-1-n}B_{2n-2}\nu_{i}T_{n}\left(\theta \right)\bar{C}$$

$$P=\text{diag}(P_{1},\cdots ,P_{n-1})$$

$$R=\text{diag}(R_{1},\cdots ,R_{n-1})$$

*Once the LMIs are solved*, *L* = *P*^−1^*R*, *K* = *L* **1**_2*n*−2×1_, *and* $\theta \in \{\theta |\theta \geq \theta_{0},\theta_{0}=\text{min}\;({\left(2L_{\varphi_{s}}\left\|P\right\|/\mu \right)}^{1/(j_{s}+1)},2)\}$.

*Proof*. Following the step in Theorem 2, estimation error dynamic (7) can be contained in set associated to $\Phi^{j_{s}}$

$$\begin{array}{cc} \dot{\tilde{e}} & =\theta \left(H+LS\right)\tilde{e}+\theta^{-n}B_{2n-2}\Delta_{\varphi }\left(\hat{x},x\right)\\ & =\theta \left(H+\nabla_{LMI}T_{n}\left(\theta \right)\bar{C}+LS\right)\tilde{e}\\ & \;+\theta^{-n}B_{2n-2}\nabla_{HG}T_{n}\left(\theta \right)C\tilde{e}\\ & \in \theta \left(H\left(\Phi^{j_{s}}\right)+LS\right)\tilde{e}+\theta^{-n}B_{2n-2}\nabla_{HG}T_{n}\left(\theta \right)C\tilde{e} \end{array}$$ (18)

For every $\nabla_{LMI}\in \Phi^{j_{s}}$, there exists inequality

$$\begin{array}{c} \nabla_{LMI}\leq \sum_{i=1}^{2^{j_{s}}}p_{i}\nu_{i},\;\sum_{i=1}^{2^{j_{s}}}p_{i}=1,\;p_{i}\geq 0 \end{array}$$ (19)

then by substituting (17) and (19) into (18), we can express the derivative of the Lyapunov function as

$$\begin{array}{cc} \dot{V} & =\theta {\tilde{e}}^{⊤}\{He(PH\left(\nabla_{LMI}\right)+RS\theta^{-1-n}PB_{2n-2}\nabla_{HG}T_{n}\left(\theta \right)\bar{C})\}e\\ & \leq \theta {\tilde{e}}^{⊤}\{He(\sum_{i=1}^{2^{j_{s}}}p_{i}PH\left(\nu_{i}\right)+RS\theta^{-1-n}PB_{2n-2}\nabla_{HG}T_{n}\left(\theta \right)\bar{C})\}e\\ & \leq \theta {\tilde{e}}^{⊤}\{He(-\frac{\mu }{2}I_{2n-2}+\theta^{-1-n}PB_{2n-2}\nabla_{HG}T_{n}\left(\theta \right)\bar{C})\}\tilde{e} \end{array}$$ (20)

Similar to Theorem 1, if ‖*P*‖ > 1, defining $\vartheta ={\left\|P\right\|}^{-1/(j_{s}+1)}\theta$, we have

$$\begin{array}{c} \left\{He(-\frac{\mu }{2}I_{2n-2}+\theta^{-1-n}{\left\|P\right\|}^{-1}PB_{2n-2}\nabla_{HG}T_{n}\left(\vartheta \right)\bar{C})\right\}\\ \leq \{He(-\frac{\mu }{2}I_{2n-2}+\theta^{-1-n}B_{2n-2}\nabla_{HG}T_{n}\left(\vartheta \right)\bar{C})\}\\ =\left[\begin{array}{ccccccc} -\mu & \cdots & 0 & 0 & \cdots & 0 & \vartheta^{-n}L_{\varphi }\\ \vdots & \ddots & \vdots & \vdots & & \vdots & \vdots \\ 0 & \cdots & -\mu & 0 & \cdots & 0 & \vartheta^{-1-j_{s}}L_{\varphi }\\ 0 & \cdots & 0 & -\mu & \cdots & 0 & 0\\ \vdots & & \vdots & \vdots & \ddots & \vdots & \vdots \\ 0 & \cdots & 0 & 0 & \cdots & -\mu & 0\\ \vartheta^{-n}L_{\varphi } & \cdots & \vartheta^{-1-j_{s}}L_{\varphi } & 0 & \cdots & 0 & -\mu \end{array}\right] \end{array}$$ (21)

When $\vartheta >{\left(\frac{2L_{\varphi }}{\mu }\right)}^{1/(1+j_{s})}$ and *θ* > 2, it follows that

$$\mu >2\vartheta^{-1-j_{s}}L_{\varphi }>\sum_{i=j_{s}+1}^{n}\vartheta^{-i}L_{\varphi }$$

and

$$\mu >\vartheta^{-j}L_{\varphi },\;\forall j\in \left\{j_{s}+1,\cdots ,n\right\}$$

Consequently, the diagonal dominance of $He(-\frac{\mu }{2}I_{2n-2}+\theta^{-1-n}PB_{2n-2}\nabla_{HG}T_{n}\left(\theta \right)\bar{C})$ is achieved, leading to

$$\dot{V}<0$$

thus asymptotic stability of both $\tilde{e}$ and *e* can be established. In the case where ‖*P*‖ < 1, the asymptotic stability can be directly obtained, thereby concluding the proof.

**Remark 3**. *The proposed method offers a significant improvement over Theorem 1 by reducing the gain to its* 1/(1 + *j*_*s*_)*th power, where* *j*_*s*_ *is an adjustable integer ranging from* 1 *to n. Furthermore, in comparison to Theorem 2, it effectively reduces the number of LMIs from* 2^n^ *to* $2^{j_{s}}$. *This advancement allows for a desirable balance between the number of LMIs that need to be solved and the desired gain magnitude, achieved through the tunable parameter* *j*_*s*_. *Notably, when j*_*s*_ = *n*, *Theorem 3 simplifies to Theorem 2, while for* *j*_*s*_ = 0, *it further simplifies to Theorem 1. These observations highlight the flexibility and versatility of the proposed method*.

We can observe from Theorem 3 that the component for $H(\Phi^{j_{s}})$ exhibits a direct relationship with the selected gain parameter *θ*. Specifically, as the gain increases, the scale of $V^{j_{s}}$ decreases, thereby leading to the more relaxed constraints of the LMIs. To overcome this challenge, we could formulate an algorithm that employs an iterative approach to solve the LMIs and subsequently solve the infimum of the gain, ensuring a stable solution. Thus we present the optimization algorithm for the Lower Power Cascade High-gain observer (LPCHGO) (3) below:

**Algorithm 1**: Parameter Optimization for Observer Matrix *K* and Gain *θ*

**1** Choose the value of *j*_*s*_ within the range of 1 to *n*, initial infimum for gain $\theta_{0}^{\left(0\right)}$, the stopping change rate of gain *d*_*θ*_, and the maximum number of tolerable infeasible solutions *n*_*f*_;

**2** Solve the Linear Matrix Inequalities (LMIs) (17) with $\theta =\theta_{0}^{\left(0\right)}$, obtaining *K*^(1)^ and $\theta_{0}^{\left(1\right)}=\theta_{0}$, regardless of the feasibility of the solution;

**3** **while** $|\theta_{0}^{\left(i\right)}-\theta_{0}^{\left(i-1\right)}|>d_{\theta }$ **do**

**4**  Solve the LMIs (17) with $\theta =\theta_{0}^{\left(i\right)}$, obtaining *K*^(*i*+1)^ and $\theta_{0}^{\left(i+1\right)}=\theta_{0}$, regardless of the feasibility of the solution

**5**  **if** *Encounter n*_*f*_ *consecutive infeasible solutions*; **then**

**6**   Go back to step 1 and reduce the value of *j*_*s*_;

**7**  **end**

**8**  *i* = *i* + 1

**9** **end**

**10** Set the observer parameter matrix as *K* = *K*^(*i* + 1)^ and the infimum for gain as $\theta_{0}=\theta_{0}^{\left(i+1\right)}$, choose a proper gain parameter *θ* > *θ*_0_ depending on the demand of convergence speed

**Remark 4**. *In the initial iterations of the algorithm, when solving step 2 with a small initial value of* $\theta_{0}^{\left(0\right)}$, *the LMIs associated with the* $H(\Phi^{j_{s}})$ *may become infeasible to solve due to the large scale of the set. However, after one or two iterations, the value of* $\theta_{0}^{\left(0\right)}$ *is optimized and adjusted to a reasonable magnitude, thereby improving the feasibility of the LMIs*.

To compare the effectiveness of this method, especially to the high-order systems, we contrasted it with the pol assignment method of the cascade high-gain observer structure (LPCHGO) [5] and standard high gain method (STDHGO) [1] across various dimensions under the same lipschitz constant (*L*_*φ*_ = 1), and the final results are presented in Table 1:

**Table 1:** Gain comparison between three HGO methods.

Dimensions	Observer	Gain (*θ*)	Observer Parameter Matrix(*K*)
3	LPCHGO/LMI	5.7169	${\left[\begin{array}{cc} 5.5117 & 8.1187\\ 21.4204 & 4.8113 \end{array}\right]}^{⊤}$
	LPCHGO/POL [5]	9023.7	${\left[\begin{array}{cc} 0.4000 & 0.4000\\ 0.0700 & 0.0171 \end{array}\right]}^{⊤}$
	STDHGO [1]	96.3091	${\left[\begin{array}{ccc} 1.6056 & 3.3547 & 1.0688 \end{array}\right]}^{⊤}$
5	LPCHGO/LMI	8.8660	${\left[\begin{array}{cccc} 20.6306 & 68.5591 & 25.9634 & 10.8859\\ 156.1187 & 99.5251 & 20.8537 & 4.2119 \end{array}\right]}^{⊤}$
	LPCHGO/POL [5]	7.5004 × 10^6^	${\left[\begin{array}{cccc} 0.6000 & 0.6000 & 0.6000 & 0.6000\\ 0.3000 & 0.1110 & 0.0485 & 0.0178 \end{array}\right]}^{⊤}$
	STDHGO [1]	256.9582	${\left[\begin{array}{ccccc} 5.7483 & 15.5407 & 18.1133 & 12.6005 & 4.2844 \end{array}\right]}^{⊤}$
7	LPCHGO/LMI	20.4353	${\left[\begin{array}{cccccc} 0.0836 & 0.8644 & 0.3433 & 0.1207 & 0.0353 & 0.0265\\ 1.4185 & 2.4005 & 0.4939 & 0.0892 & 0.0146 & 0.0054 \end{array}\right]}^{⊤}\times 10^{3}$
	LPCHGO/LMI [5]	2.5293 × 10^9^	${\left[\begin{array}{cccccc} 0.8000 & 0.8000 & 0.8000 & 0.8000 & 0.8000 & 0.8000\\ 0.7700 & 0.3173 & 0.1671 & 0.0926 & 0.0485 & 0.0198 \end{array}\right]}^{⊤}$
	STDHGO [1]	2.1588 × 10^3^	${\left[\begin{array}{ccccccc} 6.4008 & 22.2823 & 35.7186 & 39.8284 & 29.2688 & 13.6470 & 3.1483 \end{array}\right]}^{⊤}$

## 4 Application to two physical applications

In this section we will use two physical models to prove our method’s applicability and performance.

### 4.1 Single-link robot system

The first model is a common single-link robot system introduced in [21].

Following the parameter used in [7], the following equations describe the model of the single-link robot system:

$$\begin{array}{cc} {\dot{z}}_{1} & =z_{2}\\ {\dot{z}}_{2} & =\frac{\bar{K}}{J_{2}N}z_{3}-\frac{F_{2}}{J_{2}}z_{2}-\frac{K}{J_{2}}z_{1}-\frac{mgd}{J_{2}}\;\text{cos}\;\left(z_{1}\right)\\ {\dot{z}}_{3} & =z_{4}\\ {\dot{z}}_{4} & =\frac{1}{J_{1}}u+\frac{\bar{K}}{J_{1}N}z_{1}-\frac{K}{J_{2}N}z_{3}-\frac{F_{1}}{J_{1}}z_{4}\\ y & =x_{1} \end{array}$$ (22)

The control input is given by:

$$\begin{array}{cc} u & =\frac{mgdJ_{1}}{J_{2}N}-\frac{J_{1}J_{2}N}{\bar{K}}\{L^{4}c_{1}x_{1}+L^{3}c_{2}x_{2}.\\ & \;+L^{2}c_{3}(\frac{K}{J_{2}N}x_{3}-\frac{mgd}{J_{2}})+Lc_{4}\frac{K}{J_{2}N}x_{4}\} \end{array}$$

parameters of the controller are listed in Table 2. Additionally, the controller parameters are given as

$$c_{1}=4,\;c_{2}=7.91,\;c_{3}=6.026,\;c_{4}=1.716,\;L=3.$$

**Table 2:** Parameters of single-link robot system.

Symbol	Description	Value
*J*_1_	Inertia constant of actuator shaft	0.15
*F*_1_	Viscous friction constant of actuator shaft	0.15
*J*_2_	Inertia constant of the link	0.2
*F*_2_	Viscous friction constant of the link	0.15
$\bar{K}$	Elasticity constant of the spring	-0.4
*N*	Transmission gear ratio	2
*m*	Mass of the link	0.8
*d*	Position of the center of gravity of the link	0.6
*g*	Gravity acceleration	9.81

Considering the 4th-order differential observability and uniform observability of (22) on the set $\left\{Z=\{z|z_{1}\in \{-\frac{\pi }{2},\frac{\pi }{2}\}\right\}$, we can employ the following coordinate transformation to obtain a new system representation:

$$\begin{array}{cc} x_{1} & =z_{1}\\ x_{2} & =z_{2}\\ x_{3} & =\frac{\bar{K}}{J_{2}N}z_{3}-\frac{F_{2}}{J_{2}}z_{2}-\frac{K}{J_{2}}z_{1}-\frac{mgd}{J_{2}}\;\text{cos}\;\left(z_{1}\right)\\ x_{4} & =\frac{\bar{K}}{J_{2}N}z_{4}-\frac{F_{2}}{J_{2}}(\frac{\bar{K}}{J_{2}N}z_{3}-\frac{F_{2}}{J_{2}}z_{2}-\frac{K}{J_{2}}z_{1}\\ & \;-\frac{mgd}{J_{2}}\;\text{cos}\;\left(z_{1}\right))-\frac{K}{J_{2}}z_{2}+\frac{mgd}{J_{2}}z_{2}\;\text{sin}\;\left(z_{1}\right). \end{array}$$ (23)

By applying this diffeomorphism transformation, the resulting system is in the observable canonical form:

$$\begin{array}{cc} {\dot{x}}_{i} & =x_{i+1},\;i=1,2,3\\ {\dot{x}}_{4} & =\frac{20\;u}{3}-\frac{2\;x_{1}}{3}-\frac{25\;x_{2}}{12}-\frac{7\;x_{3}}{2}-\frac{17\;x_{4}}{12}\\ & \;+23.544\left[-\;\text{cos}\;(x_{1})+{x_{2}}^{2}\;\text{cos}\;\left(x_{1}\right)+x_{3}\;\text{sin}\;\left(x_{1}\right)\right]\\ & \;+15.696\;x_{2}\;\text{sin}\;(x_{1}) \end{array}$$ (24)

which is suitable for designing a High-Gain observer.

To demonstrate the effectiveness and superiority of the method proposed in this paper (LPCHGO/LMI), we conducted a comparative analysis with the original pole-assign method presented in [5] (LPCHGO/POL), an additional High-Gain Observer methods introduced in [7] (FILHGO), as well as the standard high-gain observer firstly introduced in [1].

The Lipschitz constant can be calculated as *L*_*φ*_ = 2, choosing *j*_*s*_ = 2. The parameters of the above four methods are listed in Table 3. Note that the poles used in the pole-assign method are determined as (−0.1, −0.2, −0.2, −0.3, −0.3, −0.4, −0.4, −0.5).

**Table 3:** Design parameters of three high-gain observer methods.

Observer	Gain Value	Obsrever Parameter Matrix
LPCHGO/LMI	10.3105	$K=\left[\begin{array}{cc} 11.8691 & 71.0539\\ 16.0232 & 18.9177\\ 16.9167 & 10.2755 \end{array}\right]$
LPCHGO/POL [5]	181.1170	$K=\left[\begin{array}{cc} 0.5 & 0.16\\ 0.5 & 0.0525\\ 0.5 & 0.0171 \end{array}\right]$
FILHGO [7]	120	*K* = diag([0.6451, 0.1604, 0.0263, 0.0016])
		*D* = diag([1.2515, 1.3444, 1.9926, 2.0114])
STDHGO [1]	181.9861	*K* = [6.5138, 14.8364, 12.9554, 5.6972]^⊤^

**Fig 2:**
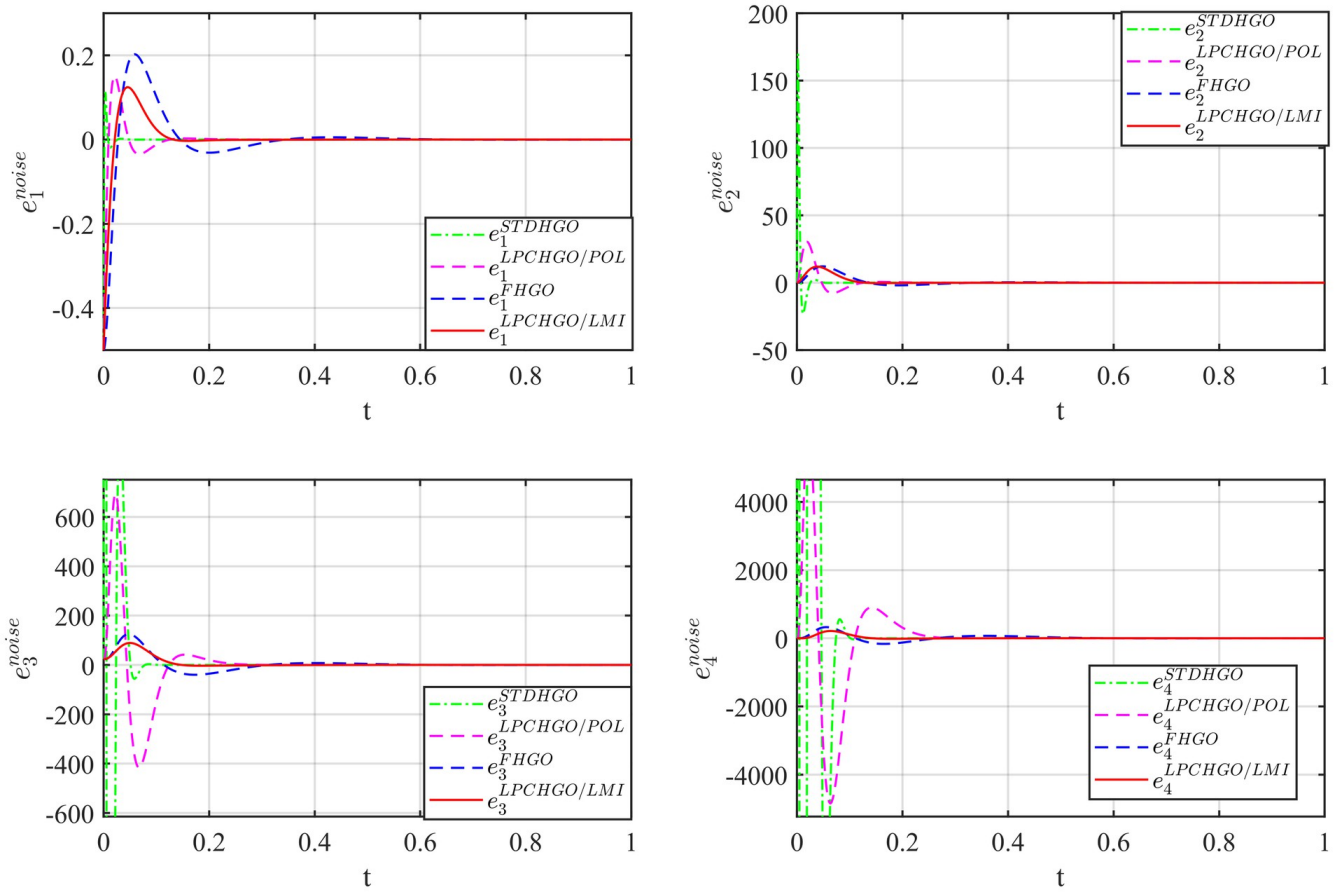
State estimation result with *v* = 0.

The initial conditions for system (22) is *x*(0) = [0.5, 0, 0, 0]^T^ and zeros for all observers, respectively. The responses of system (22) and observers with no external disturbance (*v* = 0) are shown in Fig 2. The estimated state, in particular, can track the real state accurately and quickly.

**Fig 3:**
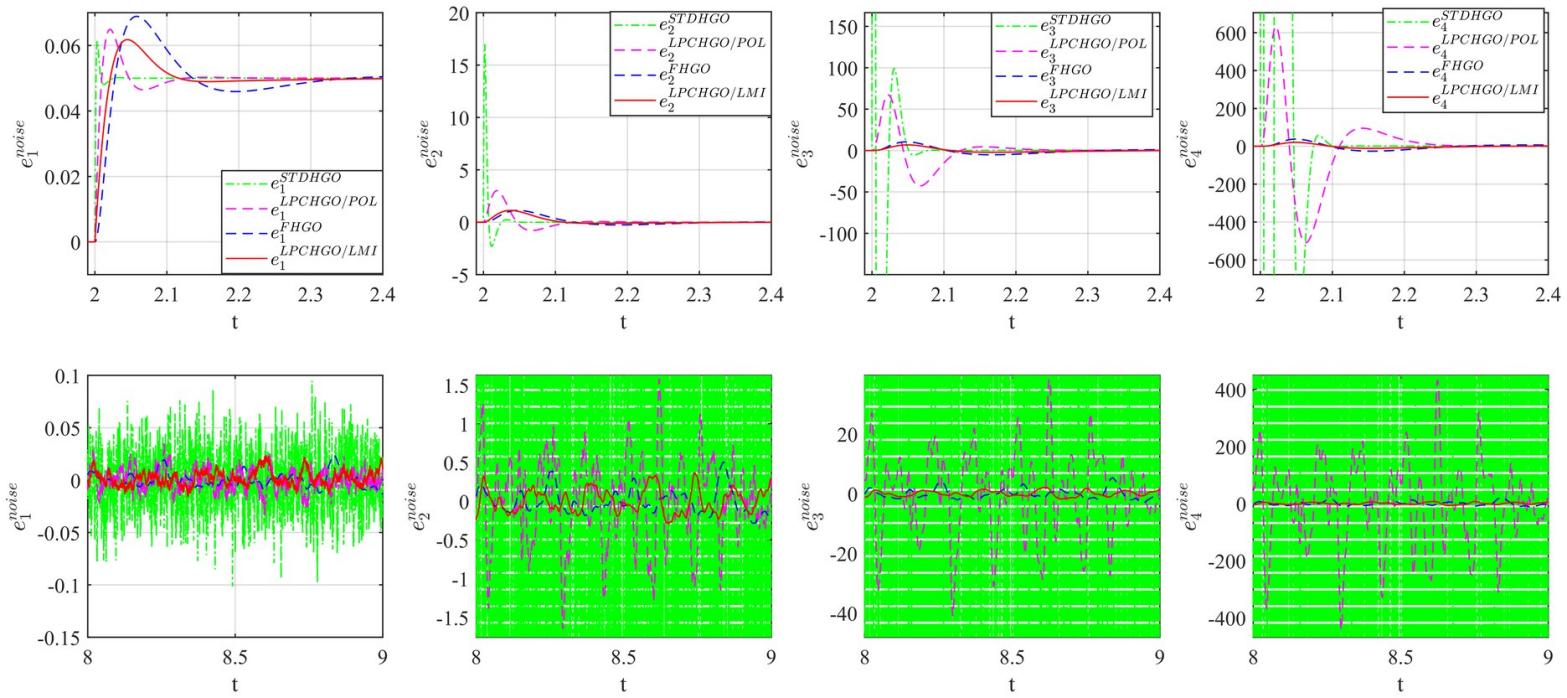
State estimation result with noise in Eq (25).

On the other hand, in Fig 3 the measurement noise is chosen as (25).

$$\begin{array}{c} v=\{\begin{array}{c} 0,\;\;\;0<t\leq 2\\ 0.05,\;\;1<t\leq 5\\ 0,\;\;\;5<t\leq 7\\ \rho (-1,1),\;\;t>7 \end{array}, \end{array}$$

where *ρ*(−1, 1)present a random noise signal bounding in the range (−1, 1).

According to the gain reduction, it can be seen clearly that the impact of measurement noise is significantly cut down.

### 4.2 Vehicle trajectory estimation

In the second example we use the vehicle trajectory estimation [22]. And the comparison between our method with the method proposed in [22] has been taken. The two-dimensional motion dynamic of the vehicle is expressed as follows:

$$\begin{array}{c} \dot{z}=\left[\begin{array}{c} \dot{X}\\ \dot{Y}\\ \dot{V}\\ \dot{A}\\ \phi \\ \beta \end{array}\right]=\left[\begin{array}{c} V\text{cos}(\phi +\beta )\\ V\text{sin}(\phi +\beta )\\ A\\ 0\\ \frac{V\text{sin}(\beta )}{l_{r}}\\ 0 \end{array}\right] \end{array}$$ (25)

where *X* and *Y* denote the two-dimensional coordinates of the vehicle’s tracking point, *V* represents the value of the tracking point’s velocity, *A* denotes the value of acceleration, *ϕ* stands for yaw angle, *β* represents the slip angle, and *l*_*r*_ signifies the distance from the front wheels to the tracking point. It is assumed that the vehicle’s acceleration and the rate of change of slip angle are nearly zero. And the canonical observable form of (25) after coordinate transforming could be expressed as below.

$$\begin{array}{c} \left\{ \begin{array}{c} \dddot{X}=-3\dddot{Y}\frac{\dddot{Y}\ddot{X}-\dddot{X}\ddot{Y}}{{\ddot{X}}^{2}+{\ddot{Y}}^{2}}+2\ddot{X}(\frac{\dddot{Y}\ddot{X}-\dddot{X}\ddot{Y}}{{\ddot{X}}^{2}+{\ddot{Y}}^{2}})\\ \dddot{Y}=3\dddot{X}\frac{\dddot{Y}\ddot{X}-\dddot{X}\ddot{Y}}{{\ddot{X}}^{2}+{\ddot{Y}}^{2}}+2\ddot{Y}(\frac{\dddot{Y}\ddot{X}-\dddot{X}\ddot{Y}}{{\ddot{X}}^{2}+{\ddot{Y}}^{2}}) \end{array}\right. \end{array}$$ (26)

Following the approach in [22], we set the yaw angle rate $\dot{\varphi }=c<1$ as a fixed value, resulting in the system parameters taking the following form:

$$\begin{array}{c} \dddot{X}=-3c\dddot{Y}+2c^{2}\ddot{X}\\ \dddot{Y}=\;3c\dddot{X}+2c^{2}\ddot{Y} \end{array}$$ (27)

**Fig 4:**
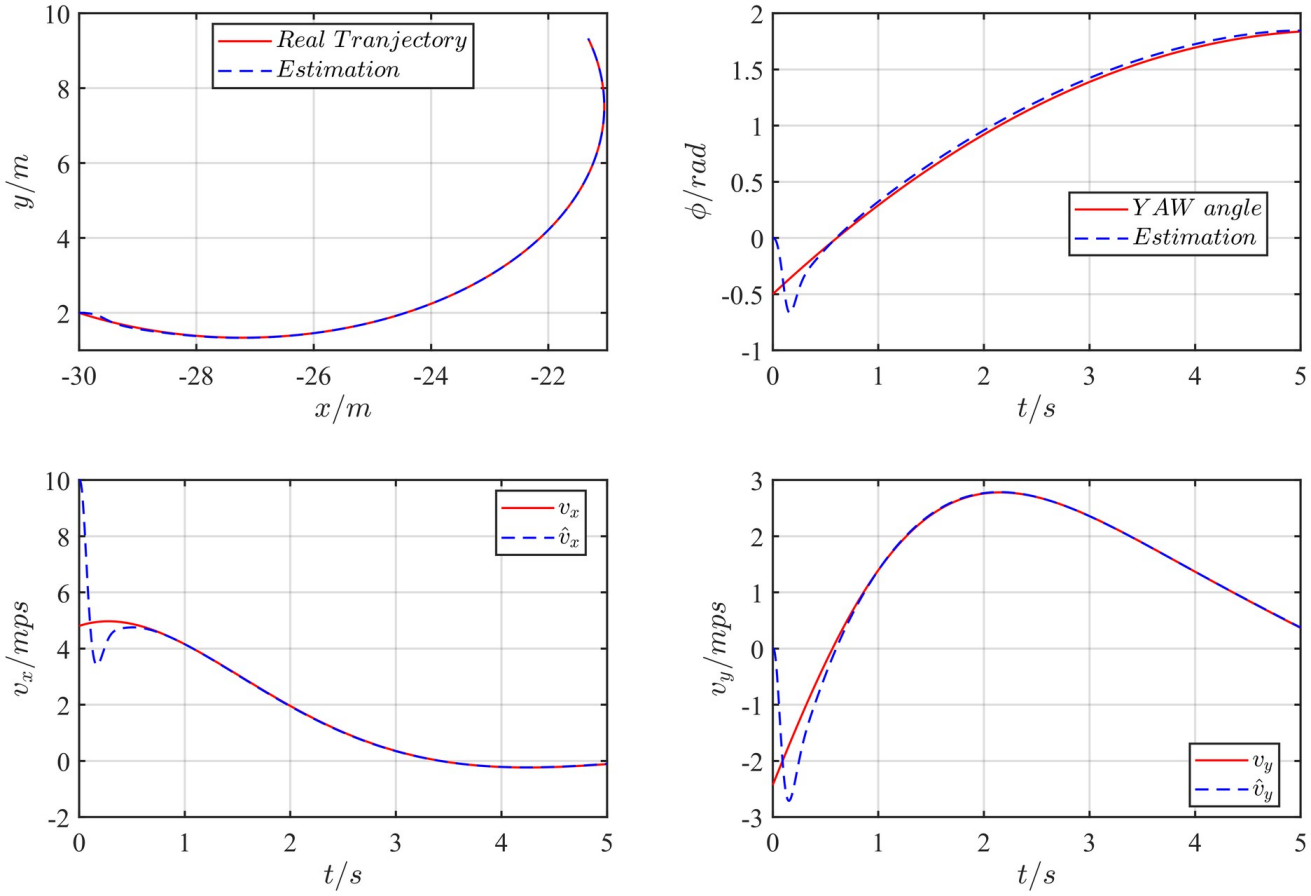
The vehicle trajectory estimation result.

Where the lipschitz constant is *L*_*φ*_ = 1.25. And the parameters of two LPCHGOs is $K=\left[\begin{array}{cc} 5.5117 & 21.4204\\ 8.1187 & 4.8113 \end{array}\right]$ and *θ* = 6.1220. Choose the initial value as *X* = −30*m*; *Y* = 2*m*; *A* = −1*m*/*s*^2^; $V=\sqrt{10^{2}+2^{2}}m/s$; *φ* = −0.5*rad*; *l*_*r*_ = 1*m*; $\dot{\phi }=10^{\circ }$. The estimation result is shown in Fig 4.

**Fig 5:**
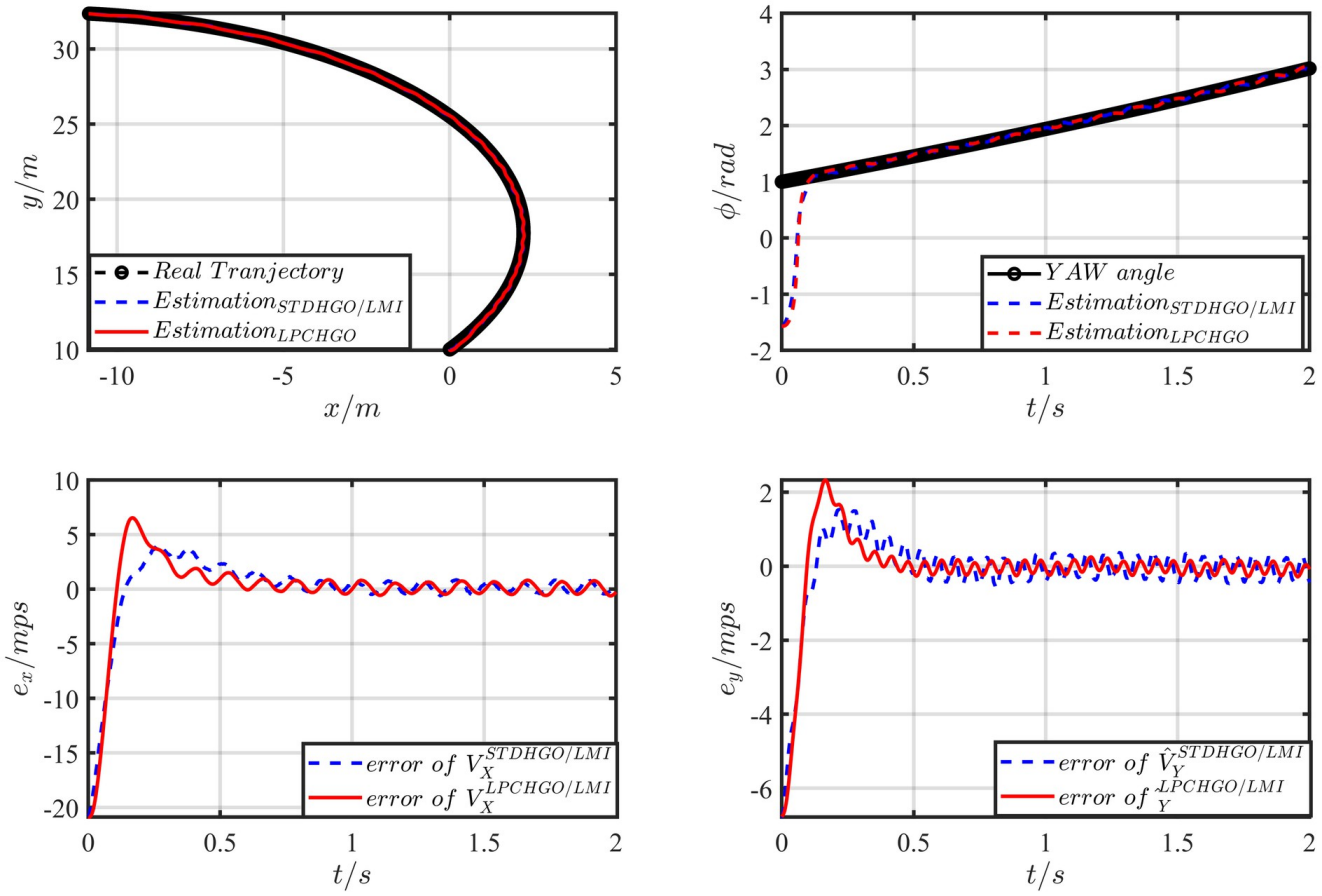
The vehicle trajectory estimation result with noise *v* = 0.5*ρ*(−0.5, 0.5) + 0.05sin(100*t*).

The comparison with the original method will be carried out in the presence of measurement noise, and the noise *v* = 0.5*ρ*(−0.5, 0.5) + 0.05sin(100*t*) is added to the positioning measurement output of the X and Y axes. The result is shown in Fig 5.

## 5 Conclusions

In this paper, we proposed an LMI/LPV method for tuning the parameters of a 2nd-order cascade HGO structure, which is proved to have a better low-pass feature than the standard structure. We could significantly reduce its gain by applying the LMI/LPV technique. The stability analysis and the simulation results could imply this fact. In the future, we could utilize the time-varying gain to improve its convergence speed.

## Supporting information

S1 File (ZIP)
